# Serum Vitamin D Metabolites by HPLC-MS/MS Combined with Differential Ion Mobility Spectrometry: Aspects of Sample Preparation without Derivatization

**DOI:** 10.3390/ijms24098111

**Published:** 2023-04-30

**Authors:** Liliia Usoltseva, Vitaliy Ioutsi, Yuriy Panov, Mariya Antsupova, Liudmila Rozhinskaya, Galina Melnichenko, Natalia Mokrysheva

**Affiliations:** Endocrinology Research Centre, 117292 Moscow, Russia

**Keywords:** vitamin D metabolites, HPLC-MS/MS, differential ion mobility

## Abstract

In current clinical practice, a thorough understanding of vitamin D metabolism is in high demand both for patients with various diseases and for healthy individuals. Analytical techniques that provide simultaneous measurement of multiple metabolites are preferred. Herein, the development of an HPLC-DMS-MS/MS method for the quantitation of vitamin D compounds (25(OH)D_3_, 25(OH)D_2_, 1,25(OH)_2_D_3_, 3-epi-25(OH)D_3_, 24,25(OH)_2_D_3_, and D_3_) in serum is described. The selected sample preparation procedure based on the combination of liquid–liquid and solid-phase extraction, which excluded a lengthy derivatization step, was compared with other common approaches. Sensitivity was increased through the implementation of differential ion mobility separation. The proposed assay allowed us to determine the low abundant 1,25(OH)_2_D_3_ with the detection limit of 10 pg/mL. The validation study showed good linearity (r^2^ > 0.99), a wide analytical range (2.5–75 ng/mL for 25(OH)D_3_), and acceptable precision (<7%) for all metabolites. The recovery ranged from 71% to 93% and the matrix effect from 0.80 to 0.95 depending on the metabolite; accuracy determination was performed using DEQAS controls.

## 1. Introduction

Growing clinical interest in the determination of vitamin D status is associated with its role not only in the regulation of calcium and phosphorus homeostasis, but also in the proper functioning of other systems, such as immune, cardiovascular, and reproductive [1]. Despite the fact that these non-classical benefits to human health are still under discussion in the medical community [2], the need for accurate measurements of vitamin D forms is undeniable [3].

Either derived from the diet or synthesized in the skin, vitamin D_3_ (cholecalciferol) is converted to 25(OH)D_3_ in the liver. Then, it is metabolized to the active form, 1,25(OH)_2_D_3_ (calcitriol), mostly in the kidneys. At the same time, 24-hydroxylated metabolites and epimers are formed [4]. In clinical practice, the main marker of vitamin D status is the major circulating form 25(OH)D_3_; however, for some disorders, all major metabolites should be quantified in order to provide proper diagnosis and therapy [5].

To assess vitamin D metabolism in patients, the most reliable technique to date is liquid chromatography–tandem mass spectrometry (LC-MS/MS). Other methods, mainly automated immunoassays, suffer inefficient vitamin D release, insufficient selectivity, and susceptibility to matrix interferences [6]. LC-MS/MS is not without drawbacks, but nonetheless, this method is considered a universal gold-standard assay for the measurement of vitamin D metabolites [7]. The determination of picogram quantities of calcitriol presents an analytical challenge that can be overcome if all stages of analysis (extraction from the biological matrix, separation from other metabolites and non-specific material, quantitation) are carefully designed. Since all metabolites in the serum are bound to the vitamin D binding protein and albumin [8], they need to be released, typically by protein precipitation (PP) via the addition of organic solvents. Subsequently, an extraction step is carried out next for further purification, generally liquid–liquid (LLE), solid-phase (SPE), or less frequently supported liquid (SLE) and salting-out-assisted liquid–liquid extraction (SALLE) [9]. Some metabolites have the same m/z transitions; therefore, complete chromatographic separation of the analytes of interest needs to be achieved [10]. Proper resolution can be attained through the implementation of reversed-phase C-18, chiral, pentafluorophenyl (PFP), and CN columns in combination with aqueous–organic mobile phases, normally water with formic acid, methanol, and acetonitrile [11]. As for detection, the most common ionization techniques are electrospray ionization (ESI) and atmospheric-pressure chemical ionization (APCI) in the positive ion mode. A common solution to improving sensitivity is the addition of a derivatization step with Cookson-type reagents [12,13] during sample preparation (SP). However, the reagents are inactive and unstable in aqueous solutions, requiring a multi-step procedure, which increases the analysis time and makes automation impossible; moreover, during the Diels–Alder reaction, two isomers are generated, so in most cases, two chromatographically resolved peaks are observed. Quite importantly, the use of derivatization reagents has detrimental effects on mass spectrometry equipment. 

It is recommended to perform quantitation using stable isotope internal standards (IS). Labeled analogs of each targeted metabolite help to correct for matrix effects and for possible procedural imprecisions and losses during sample extraction and purification [7]. The last essential aspect is the standardization of vitamin D measurement aimed at reducing inter-laboratory discrepancies. The Vitamin D External Quality Assessment Scheme (DEQAS) and certified standard reference materials are available on the market to validate designed methods.

Hence, this study aims (i) to propose a non-derivatized sample preparation optimized for 1,25(OH)_2_D_3_, 24,25(OH)_2_D_3_, 25(OH)D_3_, 25(OH)D_2_, 3-epi-25(OH)D_3_, and D_3_, and (ii) to demonstrate the advantages of using the SelexION differential mobility separation device installed between the ionization source and the vacuum interface of the mass spectrometer. We also present the analytical characteristics of the developed isotope dilution HPLC-DMS-MS/MS method, and its validation using the DEQAS (25 hydroxyvitamin and 1,25 dihydroxyvitamin D assays) controls.

## 2. Results

During method development, particular attention was paid to sample preparation and its impact on analytical results. The second phase of the study was devoted to demonstrating the advantages of differential mobility spectrometry (DMS); partial validation and external quality control are detailed below as well.

### 2.1. Principally Different Sample Preparation Approaches

Extraction of vitamin D metabolites can be achieved using different variations of the common methods, namely protein precipitation (PP) and extractions LLE, SPE, and SLE [4,7], or combinations of these techniques. However, there is practically no comprehensive information on SP optimization for the main metabolites, including D_3_ itself, which do not rely on sample derivatization [14,15,16,17].

In this work, we used an approach that helps evaluate both the matrix effects and the extraction efficiency simultaneously in order to draw conclusions quickly and clearly. In the final step, serum samples that were spiked with the analytes and processed were reconstituted in the solution of internal standards in a methanol/water mixture consistent with the mobile phase initial gradient. Therefore, quantification of the analytes of interest allows us to determine the degree of extraction, and the examination of the internal standards helps draw a conclusion on the matrix effect.

**Figure 1:**
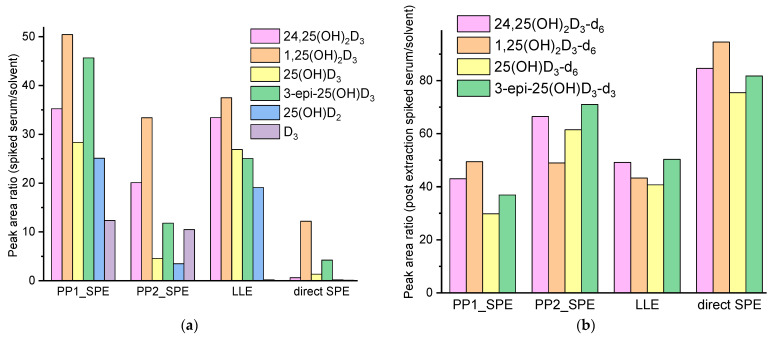
(**a**) Relative areas of the analyte peaks detected for spiked and then processed (4 types of SP) serum to the peaks of analytes in solvent (1 ng/mL in serum for all except D_3_, which had the concentration of 10 ng/mL); for D_3_, the values have been increased a hundred times for clarity. (**b**) Relative areas of the internal standards’ peaks (spiked during reconstitution) in processed serum to the peak areas of the internal standards in solvent. PP1—protein precipitation with MeOH, PP2—with MeOH and ZnSO_4_ (Procedure 3). The sample analysis technique was according to Section 4.2.

For example, consider three different SP techniques, namely protein precipitation with the help of methanol/mixture of methanol and an aqueous solution of ZnSO_4_ in combination with SPE [18], simple EtOAc LLE, and direct SPE (Agilent Bond Elut C18). The latter was included only to demonstrate our strategy. Since vitamin D metabolites are mostly bound to the vitamin D binding protein (around 85% [8]) and albumin, vitamin D extraction always includes the step to release the analytes of interest. Direct loading on an SPE cartridge, predictably, is not able to unbind free vitamin D metabolites, which is shown in Figure 1a (low extraction efficiency is observed because most analytes, while still in the bound state, did not fully adsorb to the stationary phase) and in Figure 1b (low matrix effect because eluted fraction was very pure).

The main disadvantage of the LLE procedure (especially in the case of using MTBE and EtOAc, which is not shown) is the co-extraction of lipids and other matrix constituents [19]. This was confirmed by MALDI-MS analysis of the extract (Figure S1). If no further clean-up step is undertaken, an enhanced matrix effect and mass spectrometer contamination will be observed. 

Serum precipitation with a reagent comprising ZnSO_4_ and an organic solvent, mainly methanol or acetonitrile, is a common solution for LC-MS/MS [9]. The zinc component contributes not only to the additional precipitation of proteins but also to the phospholipid removal, resulting in visually cleaner samples and reduced matrix effect (Figure 1b). Figure 1a also emphasizes the low D_3_ extraction, where none of these approaches achieved a % efficiency greater than 50%—the next section is focused on this problem.

### 2.2. Vitamin D_3_ Issue Sample Preparation Selection

Using the SP with the best performance, namely protein precipitation with MeOH and ZnSO_4_ followed by SPE (PP2_SPE), we thoroughly analyzed all fractions in terms of D_3_ content, as this analyte showed the poorest extraction efficiency, while also controlling 1,25(OH)_2_D_3_. Extracts of the loading and washing fractions and the precipitate were studied; partial evaporation of the supernatant before cartridge loading (30 and 60 min for methanol removal) was tested; moreover, to flush analytes from the stationary phase into the collection vessel, an increased volume of methanol was applied.

**Figure 2:**
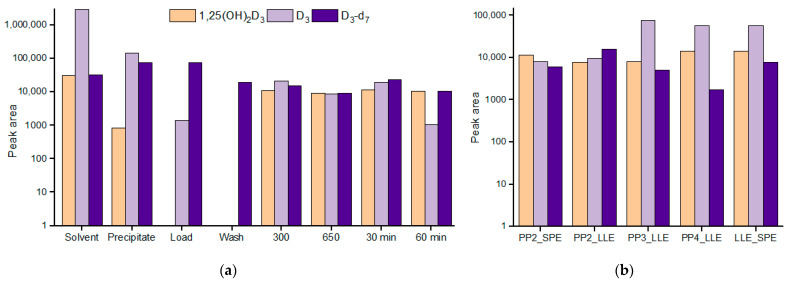
(**a**) Signals (logarithmic scale) of the analytes (100 pg/mL of 1,25(OH)_2_D_3_ and 100 ng/mL of D_3_) in the solvent and in the solutions obtained at the different stages of serum sample preparation based on protein precipitation with MeOH and ZnSO4 followed by SPE, from left to right: precipitate extract, loading fraction extract, washing fraction extract; SPE eluates obtained following two rounds of 300 µL methanol elution, and two rounds of 650 µL methanol elution; for the procedures where before loading on cartridges, the supernatants were partially evaporated during 30 or 60 min to remove methanol. (**b**) Signals of the analytes (logarithmic scale) in spiked (100 pg/mL of 1,25(OH)_2_D_3_ and 100 ng/mL of D_3_) and then processed serum. PP2_SPE and PP2_LLE—protein precipitation with MeOH and ZnSO_4_ followed either by SPE, or by LLE; PP3_LLE—protein precipitation with ACN and ZnSO_4_ followed by LLE; PP4_LLE—protein precipitation with ZnSO_4_ followed by LLE; LLE_SPE—LLE followed by SPE. For all samples, D_3_-d_7_ was spiked during reconstitution. The sample analysis technique was according to Section 4.2.

The main results are presented in Figure 2a. Firstly, the collected loading fraction contained less than 0.05% of the whole spiked D_3_ amount. Secondly, the evaporation of supernatant (after protein precipitation) in 30 and 60 min made the situation even worse, apparently, due to D_3_ sorption on the Eppendorf vial surface. Washing conditions, as shown, were well chosen—none of the analyte was detected. The increase in the elution volume did not have a significant effect, so the most critical stage was the precipitate formation. The amount of the extracted 1,25(OH)_2_D_3_ was around 2.5% and that of vitamin D_3_ was 4.8–7.4%; the data were rather scattered, which was most likely due to the incomplete and non-reproducible extraction from the sediment. Full extraction was not a goal of these experiments—the main point here was to show that in the process of protein precipitation with ZnSO_4_ and MeOH, vitamin D_3_ metabolites were lost to varying degrees, some significantly.

As a possible solution, we suggest the addition of an LLE step to the SP procedure. For example, protein precipitation with ZnSO_4_ or ZnSO_4_ and methanol, or ZnSO_4_ and acetonitrile (similarly investigated ethanol, isopropanol, and acetone which showed disappointing results), was followed by EtOAc LLE; vice versa, EtOAc LLE was performed first and followed by SPE. Looking at the data (Figure 2b), one can conclude that the most promising SP techniques are PP with ACN and ZnSO_4_ in combination with LLE (PP3_LLE) and EtOAc LLE followed by SPE. Using these techniques, calibration curves were constructed. Here, the most relevant metabolite was 1,25(OH)_2_D_3_ as the least concentrated. The achieved limits of detection (peak-to-peak S/N ≥ 3) were 10 and 25 pg/mL for the LLE_SPE procedure and PP3_LLE, respectively.

Additionally, the SALLE approach was tested. Even after cursory research, it may be concluded that SALLE is a promising approach as it is very simple and rapid; these advantages explain why recently this technique has been widely used in analytical chemistry, especially for biological samples [20]. The current conditions (Procedure 2) need to be optimized in order to increase the purity of the final solutions. Nevertheless, under the selected conditions, SALLE was characterized by the same selectivity as the PP with ACN and ZnSO_4_ in combination with LLE (limit of detection for 1,25(OH)_2_D_3_ was 25 pg/mL).

The optimized sample preparation conditions included EtOAc LLE serum extraction, followed by organic layer drying and reconstitution in a methanol/water mixture, with the resulting solution being loaded onto an SPE (C18) cartridge, washed and eluted with methanol. The eluate was evaporated to dryness, and the residue was dissolved in a methanol/water mixture and injected.

Even though the procedure has been thoroughly studied, it was associated with the use of very large (450 µL) serum volumes for modern methods. We tried to overcome this problem (Procedure 2, Figure S2) and concluded that the 10 pg/mL limit of detection for 1,25(OH)_2_D_3_ was achievable by using a reduced sample volume (300 µL, Procedure 2).

### 2.3. LLE Followed by SPE Procedure

#### 2.3.1. The Fragmentation Patterns

One of the major technical details of the present work is the use of the differential mobility separation device. Previously, this technology was successfully applied for the determination of serum estrone, estradiol, and estriol [21]. The introduction of differential ion mobility has no effect on ion fragmentation since the SelexION separation device is installed between the ionization source and the vacuum interface of the mass spectrometer, thereby affecting only the transmission of precursor ion [22,23]. 

**Figure 3:**
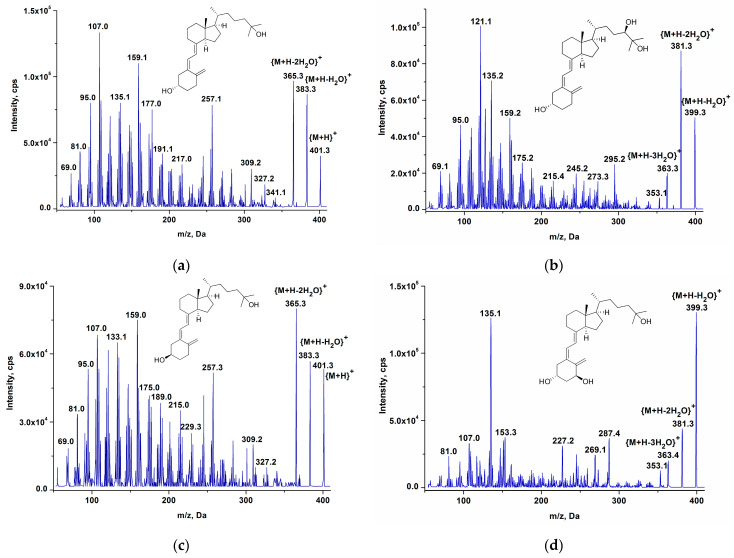
Product ion (CE = 30 V) ESI mass spectra of (**a**) 25(OH)D_3_; (**b**) 24,25(OH)_2_D_3_; (**c**) 3-epi-25(OH)D; (**d**) 1,25(OH)_2_D_3_.

Therefore, the fragmentation patterns of vitamin D and its metabolites under ion mobility ESI experimental conditions are typical for soft ionization techniques (Figure 3). Let us emphasize the limited structural information that is given by collision-induced (CID) fragmentation of the vitamin D compounds. Not without restrictions, high-resolution tandem mass spectrometry has proven to be more effective for interpreting fragmentation patterns [24]; however, for detailed chemical structure elucidation, LC-MS should be used to complement gas chromatography–mass spectrometry (GC-MS). Classic hard electron ionization used in GC-MS methods [11,25] provides additional fragment ions required for structural characterization. Undoubtedly, LC-MS is a more convenient method for the quantitative determination of non-volatile and thermolabile vitamin D compounds.

LC-MS mainly provides molecular ion information. During ESI, a slight fragmentation of the precursor ion is observed. APCI [26] and atmospheric pressure photoionization [4] may lead to more significant fragmentation of protonated molecules by the loss of neutral water molecules, thereby reducing the signal intensity and consequently the sensitivity of the method.

It should be mentioned that some vitamin D metabolites have the same precursor ions or MRM transitions that make compounds such as 25(OH)D_3_ and 3-epi-25(OH)D_3_ or 1,25(OH)_2_D_3_ and 24,25(OH)_2_D_3_ indistinguishable by mass spectrometry (Figure 3). They should be completely separated chromatographically. Differential ion mobility spectrometry is unable to separate such ions completely, but it is efficient in the separation with isobaric interfering impurities, especially in cases where chromatographic separation is ineffective. 

#### 2.3.2. The SelexION Advantage

**Figure 4:**
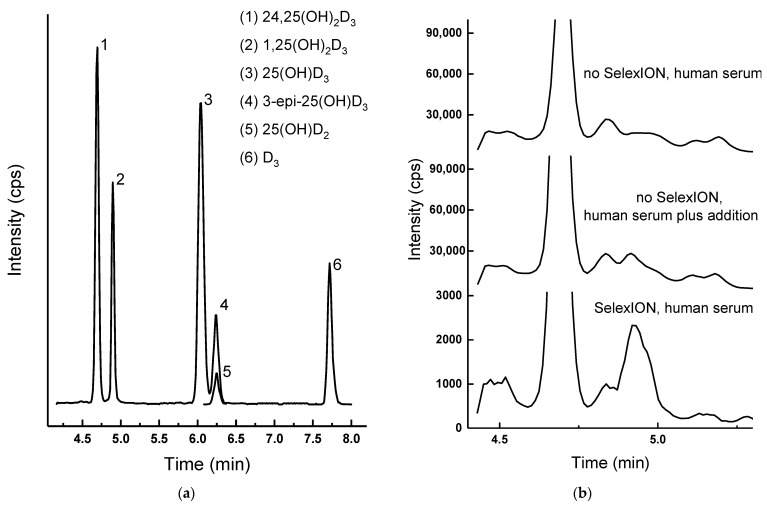
(**a**) Extracted (399.3 > 135.1, 401.3 > 383.3, 413.3 > 355.3, 385.4 > 259.3) ion chromatograms for the highest calibration point in serum (300 pg/mL 1,25(OH)_2_D_3_, see Procedures 1, 2); all Y axes are zoomed to 100% of the largest peak. (**b**) 1,25(OH)_2_D_3_ (t_R_ = 4.9 min) extracted ion chromatogram for the real sample containing 120 pg/mL of 1,25(OH)_2_D_3_, for the same sample with a standard addition (150 pg/mL spike); the detection performed with and without the SelexION system, in the latter case, the significantly increased background signal is particularly evident.

The final chromatogram, combining all main vitamin D metabolites, is presented in Figure 4a. 24,25(OH)_2_D_3_ and 1,25(OH)_2_D_3_ are separated, as well as the epimers, which became possible thanks to the addition of acetonitrile (Table 2). This is the mandatory option in routine clinical methods for the quantification of vitamin D metabolites in human serum. Meanwhile, the proposed method is actively used for these purposes [27]. The following section aims to demonstrate how this approach was developed and why it can safely be called robust. 

Without the SelexION system, the quantitative determination of metabolites is possible with the exception of the low-concentrated 1,25(OH)_2_D_3_. The limit of detection for spiked randomized charcoal-purified serum was in this case only 25 pg/mL; in fact, this is the lowest value of the reference range according to literature data [5]. On top of that, in the real samples, such concentrations were not detectable without SelexION (Figure 4b); even the sample which was additionally spiked to the total content of 1,25(OH)_2_D_3_ of 270 pg/mL was not characterized by a significant peak when SelexION was not used. Differential ion mobility spectrometry helps to circumvent many of the interferences from endogenous compounds; the signals are significantly (more than an order of magnitude) decreased, but at the same time, the signal-to-noise ratio improves.

### 2.4. LLE Followed by SPE Procedure: Partial Validation, DEQAS Controls

Method validation parameters assessed for this assay were selectivity, accuracy, precision, linearity, matrix effect, and extraction recovery. Method selectivity was verified by processing a charcoal-purified serum sample and confirming that no interfering signals were detected at the expected retention times of the vitamin D metabolites and corresponding IS. The method showed good linearity for all compounds, the determination coefficient (R^2^) values were >0.98, and the inter-day and intra-day validation results for accuracy and precision were within the guideline limits [28] for each analyte. The main validation parameters are displayed in Table 1. Cross-validation with external controls from DEQAS is presented in Figure S3; our results were all within acceptable limits. 

## 3. Discussion

It is important to understand that although numerous studies have been devoted to the LC-MS/MS determination of vitamin D metabolites in serum and plasma, the ideal assay (universal, lab-to-lab accurate and reproducible, simple and rapid), still does not exist [7]. Sample preparation, chromatography, ionization, and fragmentation vary greatly among proposed approaches [2,7,9,29] (this list is not an exhaustive one, only representative review articles are cited), and method standardization has not yet been fully established. This sophisticated task requires that all sources of error are considered and mitigated in order to obtain reliable results. 

This report presents a novel HPLC-DMS-MS/MS approach for simultaneous quantitative analysis of six vitamin D compounds that combines two new strategies: the combination of LLE and SPE extractions of analytes from serum and the improvement in sensitivity by using differential ion mobility spectrometry. This paper describes the SelexION implementation for the determination of multiple vitamin D metabolites in biological fluids. SelexION technology aims to improve the characterization and limits of quantitation for challenging samples requiring advanced analytical selectivity for the separation from isobaric species and co-eluting contaminants.

From the viewpoint of automatization, which is always an issue for high-throughput tasks, the proposed sample preparation is not the best decision. As a possible solution for making a fast, cost-effective procedure with minimum sample handling, online sample clean-up using a trap column may be evaluated in the future.

The necessity for differentiating between vitamin D metabolites which are indistinguishable in the MS imposes certain requirements for chromatographic resolution. Along with isobaric analytes, some co-eluting isobaric matrix components create difficulties in the determination of dihydroxylated products at picogram levels. The widely used derivatization stage introduces additional steps to the sample preparation and might lead to spectrometer contamination. In the established non-derivatization techniques [14,15], either some MS-unfriendly reagents were utilized or an inappropriate dynamic range was proposed. Sensitive procedures based on protein precipitation and SPE-online [16], from our point of view, may be susceptible to frequent replacement of the trap column and possible carryover. Such an outcome is less likely because the proposed chromatographic program is well-built with enough washing time with organic solvents, which in turn increases the total run time up to 35 min. Moreover, not all chromatographic systems are well equipped to use the full loop mode with a big overfill factor. 

Therefore, we suggested using the common LLE with ethyl acetate followed by ordinary C18 SPE. The main objective of this study was to show that the not-so-widespread differential mobility separation device deserves the attention of scientific groups that work with low-concentrated analytes for which the effect of mass spectral interferences, both in terms of chromatographic overlap and impact on analyte response, is very high.

The achieved analytical characteristics, such as good linearity (r^2^ > 0.99), acceptable precision (<7%), sufficient recovery (71–93%), and a low matrix effect (0.80–0.95) were within the current requirements of standard chemometrics for all the analytes [28]. The used linear ranges were 10–300 pg/mL for 1,25(OH)_2_D_3_ and 0.32–9.6, 0.48–14.4, 0.75–22.5, 2.5–75, and 12.5–375 ng/mL for 24,25(OH)_2_D_3_, 25(OH)D_2_, 3-epi-25(OH)D_3_, 25(OH)D_3_, and D_3_, respectively, which were found to be in accordance with the latest approaches [30] and clinical reference ranges [27]. Trends similar to the DEQAS measurements indicated that the suggested assay can be applied to real serum samples. The method has already been successfully used for a comprehensive study of vitamin D metabolism in patients with hypoparathyroidism [27]. 

What is important to discuss here is that an advanced understanding of the role of vitamin D in health and disease is being developed mainly thanks to the improvement of LC-MS/MS methods. However, they are still the minority in routine clinical analysis, probably due to the fact that the accurate assessment of the vitamin D spectrum has more significant implications for research than for clinical practice. At the same time, steps toward the increased demand for serum vitamin D measurements in clinical pathology laboratories are taken. 

## 4. Materials and Methods

### 4.1. Reagents and Materials

Reference standards for vitamin metabolites D_3_ (Sigma-Aldrich, Saint Louis, MO, USA), 25(OH)D_2_ (Sigma-Aldrich, Saint Louis, MO, USA), 1,25(OH)_2_D_3_ (Toronto Research Chemicals, Toronto, ON, Canada), 24,25(OH)_2_D_3_ (Toronto Research Chemicals, Toronto, ON, Canada), 25(OH)D_3_ (Toronto Research Chemicals, Toronto, ON, Canada), and 3-epi-25(OH)D_3_ (Toronto Research Chemicals, Toronto, ON, Canada) and internal standards 1,25(OH)_2_D_3_-d_6_ (Toronto Research Chemicals, Toronto, ON, Canada), 24,25(OH)_2_D_3_-d_6_ (Toronto Research Chemicals, Toronto, ON, Canada), D_3_-d_7_ (Toronto Research Chemicals, Toronto, ON, Canada), 3-epi-25(OH)D_3_-d_3_ (Eurisotop, Gif sur Yvette, France), and 25(OH)D_3_-d_6_ (Eurisotop, Gif sur Yvette, France) were used. Formic acid Optima, 99% (Fisher Chemical, Pardubice, Czech Republic), zinc sulfate monohydrate, 99% (Merck, Darmstadt, Germany), activated charcoal Norit A (Serva Feinbiochemica, Heidelberg, Germany), ammonium acetate, 96% (Merck, Darmstadt, Germany), ethyl acetate LC-MS (Fluka Analytical, Steinheim, Germany), methanol for LC-MS 99.9% (J.T. Baker, Philipsburg, NJ, USA), and acetonitrile for LC-MS, 99.9% (Honeywell, Seelze, Germany) were used. Deionized water was prepared with a Milli-Q Advantage A10 water treatment system (Millipore, Molsheim, France). Agilent Bond Elut C18 (50 mg, 1 mL) cartridges were used for SPE.

### 4.2. LC–MS/MS Analysis

An Agilent 1290 Infinity II liquid chromatography system (Agilent Technologies, Santa Clara, CA, United States) equipped with a 4-channel Flexible pump, multisampler, column thermostat, and an AB Sciex QTrap 5500 mass-spectrometer (AB Sciex, Framingham, MA, USA) with an ESI source and a SelexION differential mobility device were used. Chromatographic separation was carried out using an Acquity UPLC HSS PFP column (2.1 × 50 mm, particle size 1.8 µm, Waters), which was maintained at 40 °C. The three-component mobile phase (Table 2) was applied at a flow rate of 0.4 mL/min in gradient mode; a typical pressure trace is presented in Figure S4.

**Table 2:** Liquid chromatography gradient.

Time, Min	Acetonitrile, %	Methanol, %	Formic Acid in Water, %
0	0	50	50
1	0	50	50
1.5	0	60	40
2	0	60	40
3.2	0	70	30
4.4	3	68	29
4.8	5	70	25
5.0	5	70	25
6.0	5	75	20
6.5	0	85	15
8.0	0	85	15
8.5	0	50	50
12	0	50	50

Detection was performed in the positive ion mode, the capillary voltage was set at 5500 V, and the ion source gas pressure (GS1), turbo gas (GS2), and curtain gas pressure (CUR) were 50, 60, and 28 psi, respectively; the turbo gas temperature (TEM) was 650 °C. The parameters of the differential ion mobility system were constant for all components: the separation, compensation, offset voltages, and interface temperature were 3800 V, 6.8 V, −20 V, and 150 °C, respectively, and the carrier gas was nitrogen. A carrier gas modifier and the resolution enhancement mode were not used. Registration of the components was carried out in scheduled multiple reaction monitoring (sMRM). All MRM transitions were chosen by analyzing the product ion mass spectra of the corresponding precursor ions and optimized manually (Table 3) by ramping collision energy (CE, in the range of 10–50), declustering potential (DP, 50–200), and collision cell exit potential (CXP, 1–55). The time interval for each MRM transition was 70 s, the target scan time was 0.7 s, and the entrance potential (EP) was kept at 10 V. The generation of calibration curves for data acquisition and processing was performed using Analyst 1.6.3 software (AB Sciex). 

### 4.3. MALDI Mass Spectrometry

Matrix-assisted laser desorption/ionization (MALDI) mass spectra of the serum organic extracts were recorded on a Bruker AutoFlex II mass spectrometer time-of-flight device equipped with a N_2_ laser (337 nm, 1 ns pulse). 2,5-dihydroxybenzoic acid (DHB, 99%, Fluka) was used as the matrix. Samples were pre-mixed with the matrix solution (sample/matrix ratio was 1:10,000) and deposited onto the MALDI substrate via the “dried-droplet” technique.

### 4.4. Sample Preparation Procedures

Procedure 1. Preparation of standard solutions and calibrators. Solutions of the external and internal standards were prepared by dissolving an exact mass of individual components in ethanol or methanol in glass vials at the concentration of 1 mg/mL. Then, the working methanol solutions of the internal standards with the concentrations of 15, 200, 200, 380, and 5000 ng/mL for 1,25(OH)_2_D_3_-d_6_, 24,25(OH)_2_D_3_-d_6_, 3-epi-25(OH)D_3_-d_3_, 25(OH)D_3_-d_6_, and D_3_-d_7_, respectively, were prepared. The individual solutions of the external standards were diluted in methanol to prepare a series of mixtures for calibration and quality control (QC) samples, which were added in the amount of 10 µL to 990 µL to randomized charcoal-purified serum in accordance with the previously described procedure [31] with the adjustment of the purification being performed with heating at 57 °C and in 3–4 cycles. The purity criterion of the obtained serum was the noise level of the signal at the expected retention times for each analyte. Eight points were used for calibration at the serum concentrations of 10–300 pg/mL for 1,25(OH)_2_D_3_ and 0.32–9.6, 0.48–14.4, 0.75–22.5, 2.5–75, and 12.5–375 ng/mL for 24,25(OH)_2_D_3_, 25(OH)D_2_, 3-epi-25(OH)D_3_, 25(OH)D_3_, and D_3_, respectively. QC samples were prepared at three concentration levels: high QC level (HQC, 225 pg/mL for 1,25(OH)_2_D_3_), medium QC level (MQC, 150 pg/mL), low QC level (LQC, 30 pg/mL). All methanol solutions as well as spiked samples were stored at −80 °C and permitted to thaw for 15 min at room temperature immediately before use.

Procedure 2. Serum extraction. A 50 µL aliquot of the deuterated internal standard mixture (25(OH)D_3_-d_6_, 1,25(OH)_2_D_3_-d_6_, 3-epi-25(OH)D_3_-d_3_, 24,25(OH)_2_D_3_-d_6_, and D_3_-d_7_) was added to 300 µL of serum, vortexed, and equilibrated for 5 min. Then, 900 µL of EtOAc was added, followed by extraction (15 min) and centrifugation (14,800 rpm, 6 min, 25 °C); next, the organic layer was isolated and dried using a vacuum centrifuge (40 °C, 1350 rpm, 5 mbar vacuum). The remaining solids were reconstituted in a 4:6 methanol/water mixture (1 mL) and centrifuged, and the resulting liquid was loaded onto an Agilent Bond Elut C18 (50 mg, 1 mL) cartridge preconditioned with 0.5 mL of methanol and 1 mL of water. The cartridge was then washed with water followed by a 3:7 methanol/water mixture (1 mL of each), and analytes were eluted with 2 × 600 µL of methanol. The eluate was evaporated to dryness. A total of 115 µL of 1:1 methanol/water mixture was added to the residues; after 10 min of stirring in a shaker, samples were centrifuged (14,800 rpm, 10 min, 5 °C) and transferred to a 96-well plate.

Salting-out assisted liquid–liquid extraction approach. A 50 µL aliquot of the deuterated internal standard mixture was added to 300 µL of serum, vortexed, and equilibrated for 5 min. Then, 550 µL of ACN was added, and the sample was vortex mixed for 1 min and centrifuged (14,800 rpm, 1 min, 5 °C). Next, 300 µL of 5 M ammonium acetate was added for salting out induction, and the mixture was vortexed for 3 min and centrifuged (14,800 rpm, 2 min, 5 °C). Then, the organic layer was isolated and dried using a vacuum centrifuge. A total of 115 µL of 1:1 methanol/water mixture was added to the residues; after 10 min of stirring in a shaker, the samples were centrifuged (14,800 rpm, 10 min, 5 °C) and transferred to a 96-well plate.

Procedure 3. The comparison of protein precipitation followed by SPE, LLE, and direct SPE. The individual solutions of external standards were diluted in methanol to prepare a mixture (all 100 ng/mL, except D_3_—1000 ng/mL) which was added in the amount of 10 µL to 990 µL of randomized charcoal-purified serum to obtain a spiked serum. Protein precipitation and SPE: based on the assay procedure described previously [18], a 150 µL aliquot of 0.05 M ZnSO_4_ (PP2_SPE procedure) or H_2_O (PP1_SPE procedure) and 550 µL of MeOH were added to 300 µL of serum, followed by 15 min of vortexing and centrifugation. Then, the supernatant was loaded onto an Agilent Bond Elut C18 cartridge preconditioned with 1 mL of methanol and 1 mL of water. The cartridge was subsequently washed with water, followed by a 3:7 methanol/water mixture (1 mL of each), and the sample was eluted with 2 × 300 µL of methanol. The eluate was evaporated to dryness using a vacuum centrifuge. *LLE*: 900 µL of EtOAc was added to 300 µL of serum, followed by 15 min of vortexing and centrifugation. Then, the organic phase was evaporated to dryness. Direct SPE: 900 µL of water was added to 300 µL of the serum, and then the solution was loaded onto an Agilent Bond Elut C18 cartridge preconditioned with 1 mL of methanol and 1 mL of water. The cartridge was subsequently washed with water, followed by a 3:7 methanol/water mixture (1 mL of each), and the sample was eluted with 2 × 300 µL of methanol. The eluate was evaporated to dryness using a vacuum centrifuge. 

All residues were reconstituted in a mixture (110 µL) of internal standards in 1:1 methanol/water, and the samples were centrifuged (14,800 rpm, 10 min, 5 °C) and transferred to a 96-well plate.

Procedure 4. All stages of protein precipitation followed by the SPE procedure (D_3_ issue). The individual solutions of external standards were diluted in methanol to prepare a mixture (10 ng/mL 1,25(OH)_2_D_3_, 10 mcg/mL D_3_) which was added in the amount of 10 µL to 990 µL of randomized charcoal-purified serum to obtain a spiked serum. Then, 150 µL of 0.1 M ZnSO_4_ and 700 µL of MeOH were added to 450 µL of the spiked serum, followed by 15 min of vortexing and centrifugation (the precipitate was preserved). Then, the supernatant was loaded (loading fraction was collected) onto an Agilent Bond Elut C18 cartridge preconditioned with 1 mL methanol and 1 mL water. The cartridge was subsequently washed with 1 mL of water followed by a 1 mL 3:7 methanol/water mixture (wash solution was collected), and samples were eluted with 2 × 300 µL or with 2 × 650 µL of methanol. The eluate was evaporated to dryness using a vacuum centrifuge. To make the precipitate extract, 500 µL of water and 500 µL of EtOAc were added, followed by 30 min of vortexing and centrifugation, and then the organic phase was evaporated to dryness. To make the loading fraction extract, the solution collected during loading was first evaporated for 45 min to remove methanol, then 500 µL of EtOAc was added, followed by 15 min of vortexing and centrifugation, and then the organic phase was evaporated to dryness. To make the washing fraction extract, the solution collected during cartridge washing with 3:7 methanol/water was first evaporated for 30 min to remove methanol, then 500 µL of EtOAc was added, followed by 15 min of vortexing and centrifugation, and then the organic phase was evaporated to dryness. Methanol removal before cartridge loading: 150 µL of 0.1 M ZnSO_4_ and 700 µL of MeOH were added to 450 µL of the spiked serum, followed by 15 min of vortexing and centrifugation. Next, the supernatant was evaporated for 30 or 60 min and then loaded onto an Agilent Bond Elut C18 cartridge preconditioned with 1 mL methanol and 1 mL water. The cartridge was subsequently washed with 1 mL of water followed by a 1 mL 3:7 methanol/water mixture, and the sample was eluted with 2 × 650 µL of methanol.

All residues were reconstituted in 110 µL of D_3_-d_7_ (9 ng/mL) in 1:1 methanol/water; the samples were centrifuged (14,800 rpm, 10 min, 5 °C) and transferred to a 96-well plate.

Procedure 5. Protein precipitation followed by SPE vs. protein precipitation followed by LLE and LLE followed by SPE. Spiked serum and the sample for protein precipitation followed by SPE (PP2_SPE) were prepared as described in the previous procedure. Protein precipitation followed by LLE approaches: (1) 150 µL of 0.1 M ZnSO_4_ and 700 µL of MeOH (PP2_LLE procedure) were added to 450 µL of the spiked serum, followed by 15 min of vortexing and centrifugation. The supernatant was first evaporated for 60 min using a vacuum centrifuge (40 °C, 1350 rpm, 5 mbar vacuum) for methanol removal, and then 900 µL of EtOAc was added for a 15 min extraction followed by centrifugation; next, the organic layer was isolated and dried. (2) The PP3_LLE procedure was the same as the previous one, with the exception of 700 µL of ACN being used instead of MeOH during PP. (3) In the PP4_LLE procedure, only zinc sulfate was included—15 µL of 1.25 M ZnSO4 was added to 450 µL of the spiked serum, and the mixture was vortexed and centrifuged. Then, 900 µL of EtOAc was added to the supernatant for a 15 min extraction followed by centrifugation; next, the organic layer was separated and dried. LLE followed by SPE (LLE_SPE procedure): 900 µL of EtOAc was added to 450 µL of the spiked serum for a 15 min extraction followed by centrifugation; next, the organic layer was isolated and dried. The residues were reconstituted in a 4:6 methanol/water mixture (1 mL), centrifuged, and the resulting liquid was loaded onto an Agilent Bond Elut C18 (50 mg, 1 mL) cartridge preconditioned with 0.5 mL methanol and 1 mL water. The cartridge was then washed with water followed by a 3:7 methanol/water mixture (1 mL of each), and the analytes were eluted with 2 × 650 µL of methanol. The eluate was evaporated to dryness using a vacuum centrifuge.

All residues were reconstituted in 110 µL of D3-d_7_ (9 ng/mL) in 1:1 methanol/water, and the samples were centrifuged (14,800 rpm, 10 min, 5 °C) and transferred to a 96-well plate.

Procedure 6. Partial validation. Method validation was carried out following the ICH guideline [28]. The accuracy (assessed by comparing the QC concentrations to the nominal value) and precision (characterized by RSD%) of the method were evaluated with 5 replicates at two concentration ranges (LQC and HQC), intra-day and inter-day (on three consecutive days), according to Procedure 2. To measure the matrix effect, a blank serum was processed (6 replicates) and spiked during the final reconstitution step with the IS and analyte mixtures at the required concentration level; standard solutions at the same concentration were also prepared in water and methanol. To assess the recovery, the analyte response in QC samples (6 replicates) at two concentrations (spiked with the analyte and processed) was compared with the response in a blank randomized charcoal-purified serum (processed and then spiked with the IS and analyte mixtures during reconstitution).

## 5. Conclusions

High-performance liquid chromatography–tandem mass spectrometry equipped with a differential ion mobility device was applied for the simultaneous determination of six relevant vitamin D metabolites, including 1,25(OH)_2_D_3_, in human serum. The optimization process of the sample preparation procedure based on the combination of liquid–liquid and solid-phase extraction without a derivatization step was described in detail and compared to other common procedures. The final assay was validated, standardized, and applied to the analysis of real samples. This study highlights new strategies and thus forms the basis not only for vitamin D analysis (for instance, the scope of metabolites can be widened) but also for other multi-metabolite-profiling HPLC-DMS-MS/MS methods.

## Figures and Tables

**Table 1:** The most relevant parameters of partial method validation.

Analyte	Concentration, ng/mL ^1^	Inter-Day Precision, %	Accuracy, %	IS Normalized MF ^2^ (% CV)	Analyte Recovery, % (% CV)	IS Recovery, % (% CV)
1,25(OH)_2_D_3_	30	7.0	87.5	1.02 (9.7)	95 (6.5)	71 (2.3)
	225	3.6	95.9	0.90 (11.1)	90 (7.7)	73 (5.0)
24,25(OH)_2_D_3_	0.96	2.2	92.8	0.91 (2.0)	79 (4.5)	66 (4.0)
	7.2	3.7	92.2	0.99 (10.5)	84 (3.8)	70 (3.9)
25(OH)D_3_	7.5	3.5	95.4	0.89 (5.2)	78 (5.9)	67 (5.3)
	56.3	1.6	113.0	0.89 (10.2)	82 (5.7)	65 (5.2)
3-epi-25(OH)D_3_	2.25	3.7	89.6	0.94 (6.9)	82 (7.2)	68 (7.5)
	16.9	1.4	96.8	0.90 (10.4)	76 (7.8)	61 (8.8)
25(OH)D_2_	1.4	6.1	95.6	0.81 (3.8)	77 (8.8)	67 (5.3) ^3^
	10.8	5.9	100.8	0.79 (10.0)	81 (10.1)	65 (5.2) ^3^
D_3_	37.5	5.5	90.2	0.98 (13.6)	70 (12.4)	62 (10.0)
	281	4.1	91.0	1.07 (9.3)	72 (9.0)	58 (9.2)

^1^ pg/mL for 1,25(OH)_2_D_3_. ^2^ Matrix effect. ^3^ 25(OH)D_3_-d_6_ as the IS.

**Table 3:** MRM transitions used for detection of the vitamin D metabolites.

Analyte	Transition Type	Q1	Q3	t_R_, min	CE, V	DP, V	CXP, V
1,25(OH)_2_D_3_	quantifier	399.3	135.1	4.9	28	89	16
	qualifier	399.3	381.3		19	89	14
24,25(OH)_2_D_3_	quantifier	417.3	399.3	4.7	13	66	15
	qualifier	417.3	381.3		15	66	14
25(OH)D_3_	quantifier	401.3	383.3	6.1	13	59	15
	qualifier	401.3	365.4		17	59	13
3-epi-25(OH)D_3_	quantifier	401.29	383.2	6.3	14	110	9
	qualifier	401.29	365.3		17	110	9
25(OH)D_2_	quantifier	413.3	355.3	6.3	15	110	7
	qualifier	413.3	395.3		13	110	7
D_3_	quantifier	385.4	259.3	7.7	20	100	30
	qualifier	385.4	159.2		32	100	18
1,25(OH)_2_D_3_-d_6_	IS	405.3	135.0	4.9	30	170	12
24,25(OH)_2_D_3_-d_6_	IS	423.3	387.5	4.7	16	150	7
25(OH)D_3_-d_6_	IS	407.4	389.3	6.1	12	120	11
3-epi-25(OH)D_3_-d_3_	IS	404.4	368.3	6.3	18	150	6
D_3_-d_7_	IS	392.4	266.3	7.7	20	90	30

## Data Availability

The datasets generated and/or analyzed during the current study are available from the corresponding author upon reasonable request. The data are not publicly available due to due to privacy reasons.

## Supplementary Materials

The following supporting information can be downloaded at: https://www.mdpi.com/article/10.3390/ijms24098111/s1.
