# Supplementary material for: Serum Vitamin D Metabolites by HPLC-MS/MS Combined with Differential Ion Mobility Spectrometry: Aspects of Sample Preparation without Derivatization
Source: Int J Mol Sci. 2023 Apr 30;24(9):8111. doi: 10.3390/ijms24098111 (PMC10179168; doi:10.3390/ijms24098111)
Supplement: Supplementary file 1 [file ijms-24-08111-s001.zip › ijms-2367399-supplementary.pdf]

Supporting Information

Serum vitamin D metabolites by HPLC-MS/MS combined with differential ion mobility spectrometry: Aspects of sample preparation without derivatization

by

Liliia Usoltseva*, Vitaliy Ioutsi, Yuriy Panov, Mariya Antsupova, Liudmila Rozhinskaya, Galina Melnichenko and Natalia Mokrysheva

Endocrinology Research Centre, 117292 Moscow, Russia

* Corresponding author Liliia Usoltseva. E-mail address: usoltseva.liliya@endocrincentr.ru

All peaks in the MALDI mass spectrum of the extract sample were assigned to the phospholipids and triglycerides species. The assignment was performed using data reported earlier (Table S1) [1].

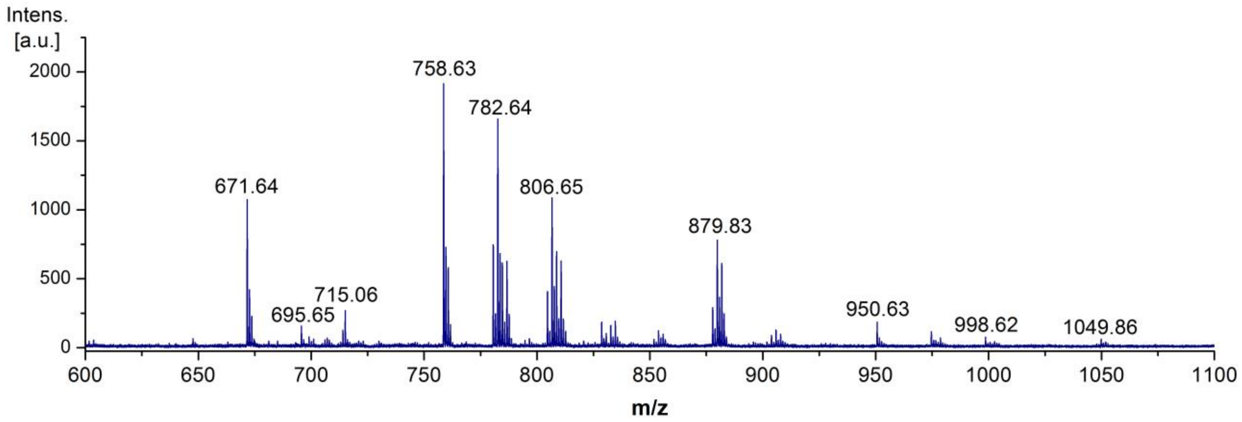

Figure S1. MALDI mass spectrum of the EtOAc serum extract (DHB).

Table S1. Assignment of the MALDI mass-spectrum peaks.

	m/z	Ion	Species	Chain length
1	671.64	$\{M+H\}^+$	SM	18:2/14:1
2	695.65	$\{M+Na\}^+$	SM	18:1/14:1
3	758.63	$\{M+H\}^+$	PC	16:0/18:2
4	760.66	$\{M+H\}^+$	PC	16:0/18:1
5	780.62	$\{M+Na\}^+$	PC	16:0/18:2
6	782.64	$\{M+Na\}^+$	PC	16:0/18:1
7	784.66	$\{M+H\}^+$	PC	18:0/18:3
8	786.67	$\{M+H\}^+$	PC	18:0/18:2
9	804.63	$\{M+Na\}^+$	PC	16:0/20:4
10	806.65	$\{M+Na\}^+$	PC	18:0/18:3
11	808.66	$\{M+Na\}^+$	PC	18:0/18:2
12	828.64	$\{M+H\}^+$	PC	18:0/21:2
13	830.65	$\{M+H\}^+$	PC	18:0/21:1
14	832.66	$\{M+Na\}^+$	PC	18:0/20:4
15	834.68	$\{M+Na\}^+$	PC	18:0/20:3
16	851.79	$\{M+Na\}^+$	TG	16:0/16:0/18:3
17	853.80	$\{M+Na\}^+$	TG	16:0/16:0/18:2
18	855.83	$\{M+Na\}^+$	TG	16:0/16:0/18:1
19	877.81	$\{M+Na\}^+$	TG	16:0/18:2/18:2
20	879.83	$\{M+Na\}^+$	TG	16:0/18:1/18:2
21	881.85	$\{M+Na\}^+$	TG	16:0/18:1/18:1
22	901.81	$\{M+Na\}^+$	TG	18:2/18:2/18:2
23	903.84	$\{M+Na\}^+$	TG	18:1/18:2/18:2
24	905.85	$\{M+Na\}^+$	TG	18:0/18:2/18:2
25	907.87	$\{M+Na\}^+$	TG	18:0/18:1/18:2
26	1049.86	$\{M+Na\}^+$	TG	20:0/20:1/24:2

*SM – sphingomyeline, PC – phosphatidylcholine, TG – triglyceride.

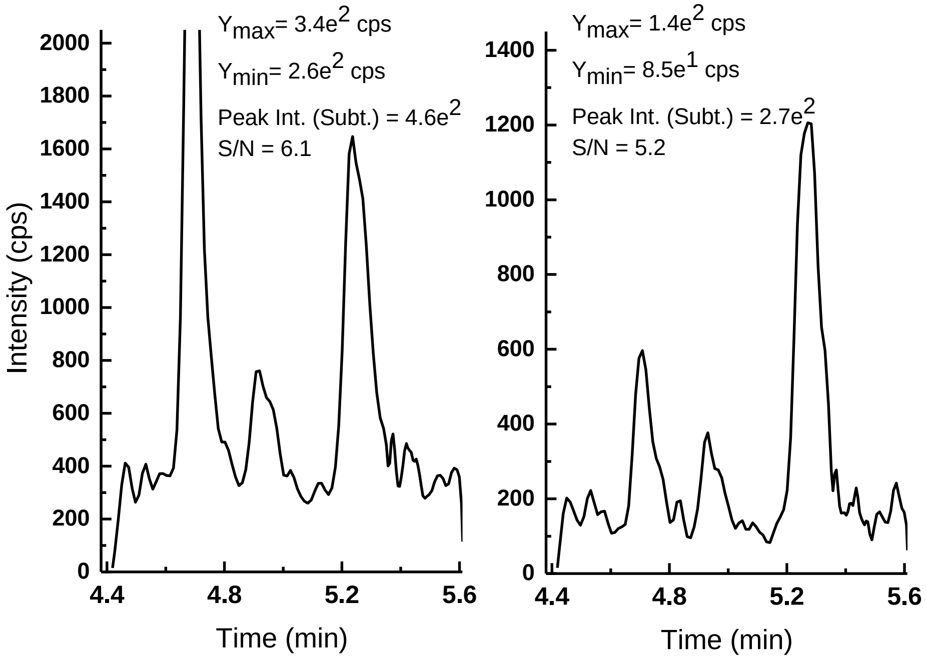

Figure S2. **1,25(OH)$_2$D$_3$ (t_R=4.9 min) extracted ion chromatograms at the limit of detection level (10 pg/ml in serum) for LLE followed by SPE procedure using 450 (left) and 300 µl (right) of serum, Peak to Peak S/N ratios were 6.1 and 5.2, respectively.**

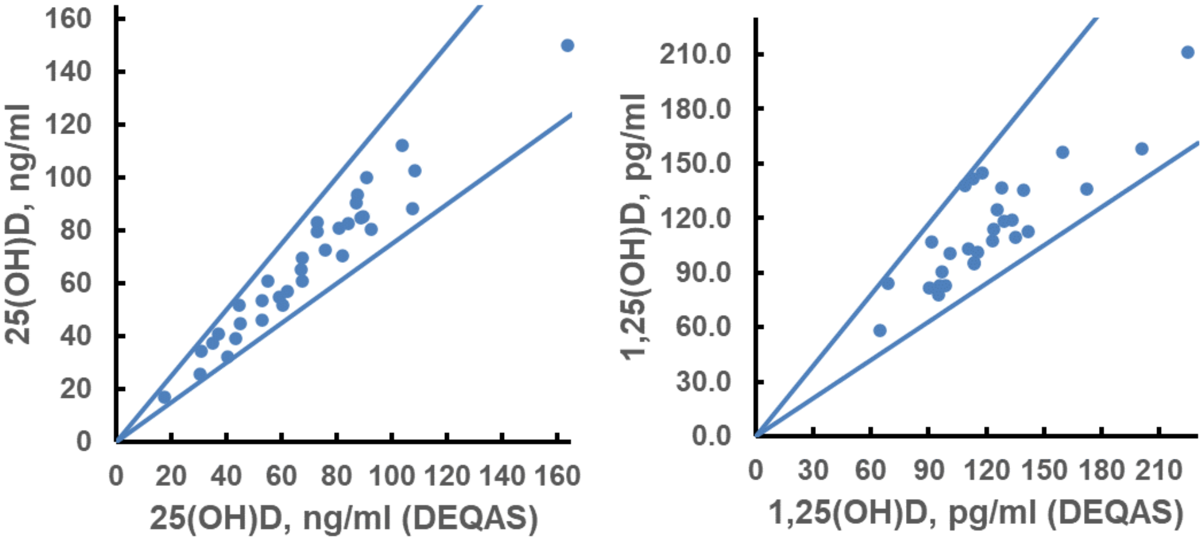

Figure S3. Comparison between DEQAS data for 1,25(OH)D and 25(OH)D scheme and our lab results. Area between the lines indicates DEQAS acceptable range (±30% and ±25% from the target value).

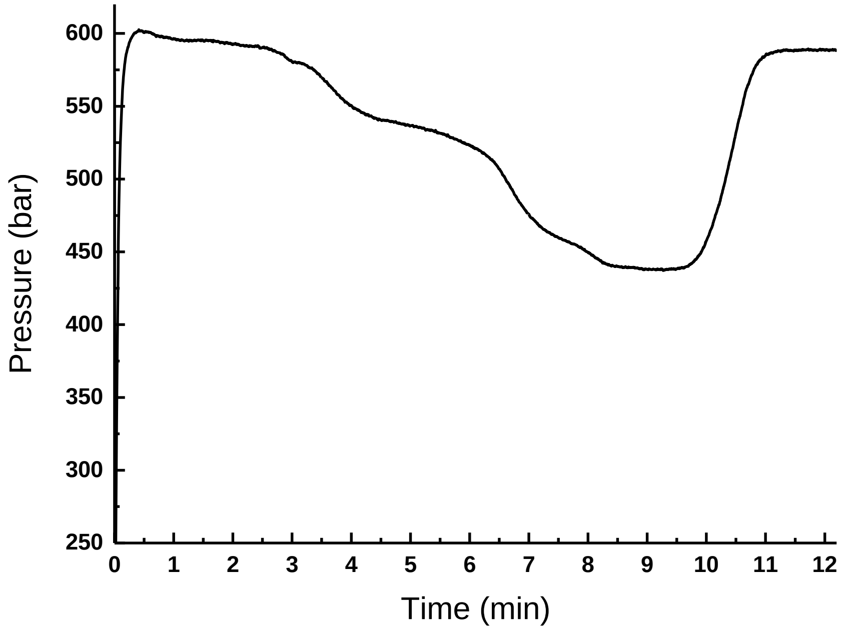

Figure S4. A typical pressure trace for proposed liquid chromatography gradient (Table 2).

References

1. Hori, A.; Yamashita, M.; Yamaura, M.; Hongo, M.; Honda, T.; Hidaka, H. Rapid Quantitative Analysis of Human Serum Sphingomyelin Species Using MALDI-TOF Mass Spectrometry with Lipid Hydrolase Treatment. *Clinica Chimica Acta* **2016**, *453*, 95–99, doi:10.1016/j.cca.2015.09.009.
